# The Expression of Dress

**Published:** 1864-06

**Authors:** 


**The Expression of Dress.**—Women are more like flowers than we think. In their dress and adornment they express their natures, as the flowers do in their petals and colors. Some women are like the modest daisies and violets—they never look or feel better than when dressed in a morning wrapper. Others are not themselves unless they can flame out in gorgeous dyes, like the tulip or the blush-rose. Who has not seen women just like white lilies? We know several double marigolds and poppies. There are women fit only for velvets, like the dahlias; others are graceful and airy, like azaleas. Now and then, you see hollyhocks and sunflowers. When women are free to dress as they like, uncontrolled by others, and not limited by their circumstances, they do not fail to express their true characters, and dress becomes a form of expression very genuine and useful.—*Meredith.*

